# Effectiveness of a smartphone app in increasing physical activity amongst male adults: a randomised controlled trial

**DOI:** 10.1186/s12889-016-3593-9

**Published:** 2016-09-02

**Authors:** Tim Harries, Parisa Eslambolchilar, Ruth Rettie, Chris Stride, Simon Walton, Hugo C. van Woerden

**Affiliations:** 1 Kingston Business School, Kingston Hill Campus, Kingston upon Thames, KT2 7LB United Kingdom; 2 Swansea University, Singleton Park, Swansea, SA2 8PP United Kingdom; 3 IWP, University of Sheffield, Conduit Road, Sheffield, South Yorkshire S10 1FL United Kingdom; 4 Oxford e-Research Centre, 7 Keble Road, Oxford, Oxon OX1 3QG United Kingdom; 5 NHS Highland, Assynt House, Beechwood Park, Inverness, IV2 3BW United Kingdom; 6 University of the Highlands and Islands, Centre for Health Sciences, Inverness, IV2 3JH United Kingdom

**Keywords:** Physical activity, RCT, Smartphone, Males

## Abstract

**Background:**

Smartphones are ideal for promoting physical activity in those with little intrinsic motivation for exercise. This study tested three hypotheses: H1 – receipt of social feedback generates higher step-counts than receipt of no feedback; H2 – receipt of social feedback generates higher step-counts than only receiving feedback on one’s own walking; H3 – receipt of feedback on one’s own walking generates higher step-counts than no feedback (H3).

**Methods:**

A parallel group randomised controlled trial measured the impact of feedback on steps-counts. Healthy male participants (*n* = 165) aged 18–40 were given phones pre-installed with an app that recorded steps continuously, without the need for user activation. Participants carried these with them as their main phones for a two-week run-in and six-week trial. Randomisation was to three groups: no feedback (control); personal feedback on step-counts; group feedback comparing step-counts against those taken by others in their group. The primary outcome measure, steps per day, was assessed using longitudinal multilevel regression analysis. Control variables included attitude to physical activity and perceived barriers to physical activity.

**Results:**

Fifty-five participants were allocated to each group; 152 completed the study and were included in the analysis: *n =* 49, no feedback; *n =* 53, individual feedback; *n =* 50, individual and social feedback. The study provided support for H1 and H3 but not H2. Receipt of either form of feedback explained 7.7 % of between-subject variability in step-count (F = 6.626, *p* < 0.0005). Compared to the control, the expected step-count for the individual feedback group was 60 % higher (effect on log step-count = 0.474, 95 % CI = 0.166–0.782) and that for the social feedback group, 69 % higher (effect on log step-count = 0.526, 95 % CI = 0.212–0.840). The difference between the two feedback groups (individual vs social feedback) was not statistically significant.

**Conclusions:**

Always-on smartphone apps that provide step-counts can increase physical activity in young to early-middle-aged men but the provision of social feedback has no apparent incremental impact. This approach may be particularly suitable for inactive people with low levels of physical activity; it should now be tested with this population.

## Background

This study used a randomised controlled trial to assess the impacts on the physical activity of healthy male adults of using smartphones to provide conventional and social norms feedback on their day-to-day walking. Most existing apps are aimed at people with an interest in sport or exercise; they require high levels of commitment and investment and a willingness to self-identify as exercise-oriented. This study tested an intervention aimed at people who might have little intrinsic motivation to increase their physical activity but would benefit from engagement of their curiosity about their own lives and new awareness of the hidden physical activity that was already of their everyday lives.

Walking is one of the most widely available types of physical activity and is linked with lower rates of mortality [1]. It does not require special skills, locations or equipment, is often a natural part of domestic and work routines and is described by most people as enjoyable and relaxing [2]. As a means of achieving greater health through physical activity, it is available to all those with the necessary physical mobility and is “readily repeatable, self-reinforcing and habit-forming” [3].

Walking is highly beneficial for health [3, 4]. It can prevent or ameliorate long-term conditions such as obesity, type-2 diabetes and cardiovascular disease [5, 6]; it helps reduce depression and anxiety, can enhance self-esteem [7–9] and has been shown to reduce cognitive decline [10].

Smartphones and their embedded computer technologies are increasingly being used to promote physical activity [11–14]. Seventy-five percent of the UK population owns a smartphone [15] and nearly 9 in 10 of these have at some time downloaded an app [16]. Indeed, so-called *fitness apps* now comprise 21 % of the UK’s downloaded apps [17].

Some apps allow users to compare their own data with that of other users [18–21]. One way of using such data, the *social norms approach*, relies on the tendency for people to seek to conform to what they perceive to be the normal behaviour of others. This approach has been successfully used to influence behaviour in fields as diverse as alcohol abuse, sexual behaviour, the payment of unpaid tax and domestic electricity consumption [14]. A social norms approach has not been evaluated in a smartphone app for promoting physical activity; nor have previous studies compared the social norms approach with the use of individual feedback alone in the context of physical activity apps.

The trial set out to test three hypotheses. Theories about the effects of social norms on behaviour [22] and evidence from the use of the social norms approach in other domains [23] led us to expect that those receiving social feedback would have higher step-counts than those who did not receive any feedback:

- H1: those with access to social feedback will have higher step-counts than those receiving no feedback

Few previous studies of the social norms approach have controlled for the personal feedback that is implicit in any attempt to compare people’s behaviour to an average or norm. To separate out the effect of the social comparison from the effect of the feedback on people’s own activity, we included a second treatment that provided participants with personal feedback but not comparative data from a peer group. We anticipated that those in this individual feedback group would show a higher step-count than the control group but a lower step count than the social norms group. We hypothesised that:

- H2: those receiving social norms feedback will have higher step-counts than those that only receive feedback on their own walking

Most previous studies indicate that feedback on a person’s own physical activity levels is itself sufficient to prompt increased walking. We therefore hypothesised that:

- H3: those only receiving feedback on their own walking will have higher step-counts than those receiving no feedback

## Methods

The study used a randomised controlled trial design to test the effectiveness of using this app amongst men aged 22–40 years. The intervention consisted of an app and a series of automated emails (see [14] for details). Although interventions that include extensive face-to-face support can be effective, they are expensive and resource intensive. We wanted to test an intervention that could be implemented on a large scale at a low cost per-capita and would therefore be suitable as a public health intervention. The key features of the app are described below, along with details of the organisation of the trial.

To remove the effects of the variability between different hardware and software platforms, the app was installed on identical phones and these were provided to the participants, who had to agree to put their Subscriber Identification Module (SIM) cards into the study phones and use them as their main mobile phones for the duration of the study. Participants were advised that the likely data usage of the app during the trial would be 20 megabytes and were informed that they would be liable for any extra data charges if they went abroad and activated the roaming function. As an incentive to participate, they were told they would keep the phones when the study was over.

The design of the version of the app provided to the two treatment groups was distinct from previous apps in three key ways. First, there was no requirement for additional equipment such as pedometers or foot pods and no need for data entry. This makes the app more attractive to those who are ambivalent about the benefits of measurement or about their ability to become fitter and healthier [24]. Secondly, while most other apps (e.g., MapMyWALK) only activate when users provide notice that they are about to begin an exercise event, this app measured activity continually and without the need for any user action. This feature of the app was intended to reduce the initial investment of time and effort, increase participation and reduce the dropout rate. In addition, it ensured that the app measured the physical activity inherent in routine, everyday activities, as well as more purposeful exercise. The third difference was that the formal goal-setting, training and coaching seen in many other apps was replaced by self-generated, informal targets that resulted from users’ engagement with the feedback. In fact, the only action required of users was that they occasionally brought the app to the foreground by clicking on the bActive icon; this was prompted by the presence of an icon on the phone screen and by regular text messages.

For such apps, measurement accuracy is now considered less important than previously and the emphasis, instead, is on the design features of the app. Early research into pedometers emphasised the importance of measurement validation using gold standard methods such as calorie expenditure and oxygen consumption [24–26]. Now, however, a lower standard of accuracy is generally accepted for apps aimed at influencing behaviour and emphasis is placed on interactive features such as goal-setting [24, 27, 28], behavioural feedback loops [11, 28–30] and features that combine motivation with enjoyment [28, 29].

Participants for this study were recruited in September 2011 by a team of 12 recruiters who approached people of approximately the age-group targeted for the study, 22–40 years, in public spaces around shopping centres in Bristol. If potential participants expressed an interest, the researchers took them through a screening questionnaire that confirmed their suitability in terms of age and their residence within easy access of the technical support team (in case of the need for technical support). To avoid the confounding effect of some participants being unable to use a mobile phone, participants also had to have an existing mobile phone contract. Potential participants were also excluded from the research if their responses to the Physical Activity Readiness Questionnaire [31] indicated that an increase in physical activity levels might be deleterious to their health.

Recruitment was limited to males. Research into motivational factors for health behaviours often attracts more female than male participants; this study was designed to help redress the resulting imbalance in much of the literature. The focus on males was also prompted by the need to carry the study phone in a pocket and concerns that women’s clothing would more often lack suitable pockets.

To confirm their commitment to the project and ensure an accurate record of electronic contact details, those who met the entry requirements and gave their consent to participate were asked to send a short text message and email message to the research team and to complete an online questionnaire. The first 165 individuals to perform these three tasks were all included as participants. The questionnaire collected data on demography and potentially confounding variables such as prior use of a smartphone, pattern of physical activity, attitude to physical activity, perceived barriers to physical activity, experience of using a smartphone, and perceived impact of the trial. A similar questionnaire at the end of the study gathered data about the experience of participation and perceived impacts. Both surveys were administered using the Qualtrics online survey platform. The results of these qualitative components of the study are not presented in this paper.

Participants were randomly assigned to one of three groups: a control (no feedback and no access to the interactive elements of the app); an individual feedback group (feedback on the participant’s own steps), and a social feedback group (feedback on the participant’s own steps and on the average steps taken by others in their group). To ensure random allocation of participants, they were listed in the order in which they had been recruited and each third participant in the list was allocated to one of the three groups. This process was undertaken manually by a research-team member who had not had any contact with the participants, and was therefore blinded in relation to other details of the participants. Participants were blinded in that all three groups had a similar looking icon on their phone, although access to the data in the app was not visible to participants in the control group.

In studies using multi-level analysis methods, sample size calculations are highly complex and should be used with caution [32]. In this study, a power analysis was also precluded by the absence of evidence on the likely effect-size. In addition, the cost of the phones we were supplying to participants limited the number of participants. However, we ensured that the resources available for purchasing the study phones allowed us to achieve a sample size that exceeded the published recommendation of at least 50 participants for each factor being considered [33].

Prior to the start of the trial, participants from all three groups were provided, via courier, with the study phone and instructions on how to use it and how to insert a SIM card. The app was disabled until the start of the trial, when it was remotely enabled, presented itself to the user and, in the case of the two treatment groups, offered a guide to its use. Subjects were told that the aim of the study was to measure the amount of walking they did; only those in the treatment groups received overt encouragement to increase their walking.

**Fig. 1:**
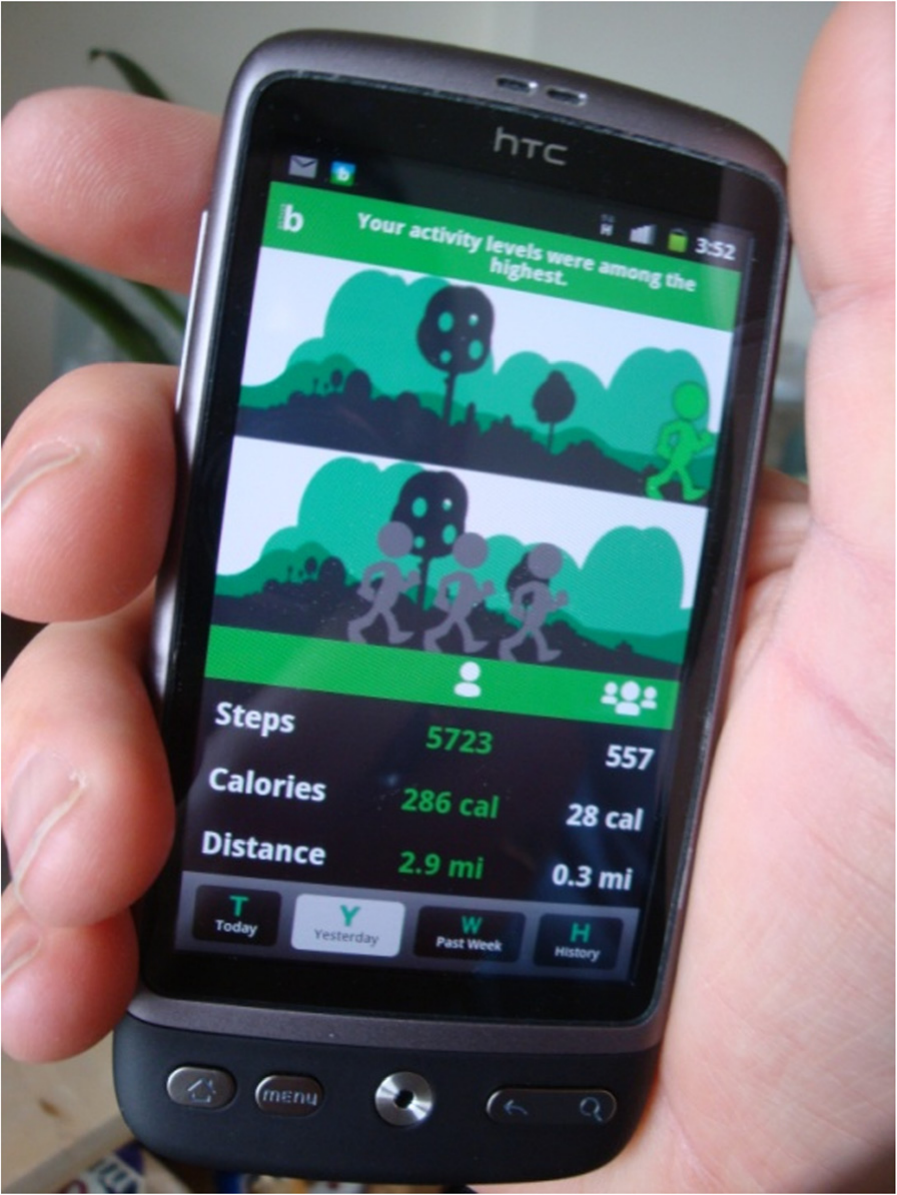
The bActive app. [This is a picture of a hand holding a mobile phone]

**Fig. 2:**
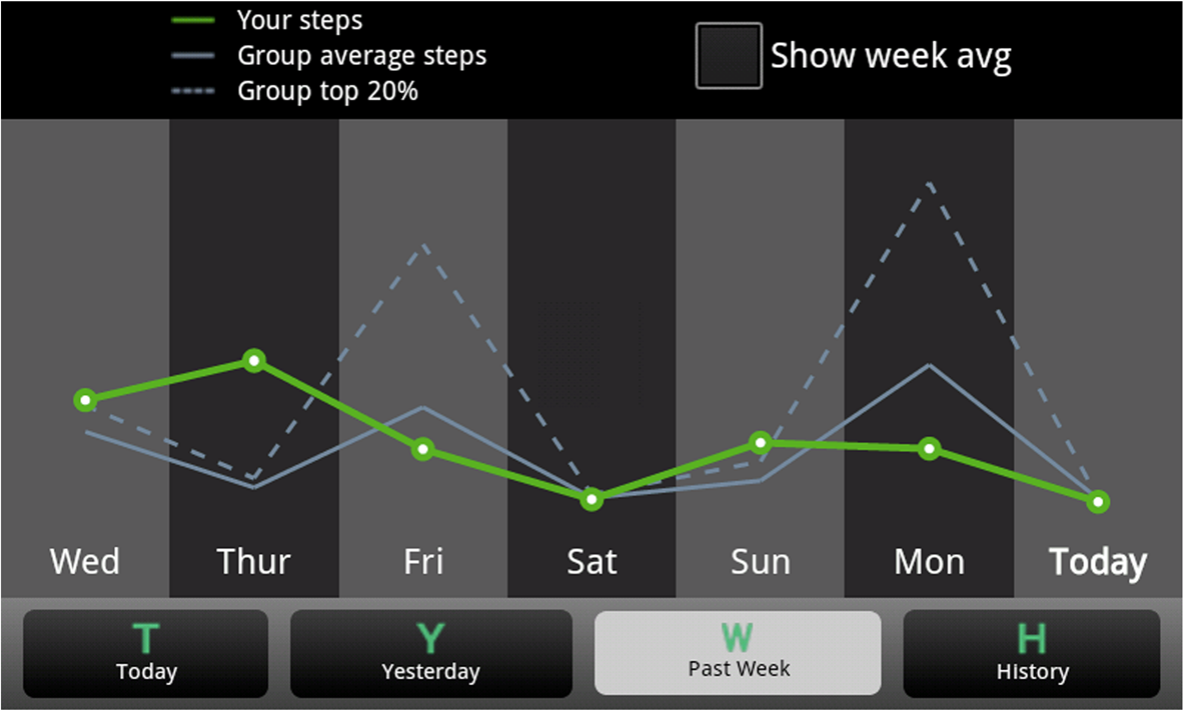
The Today, Yesterday, and Past Week screens. [This image includes three photographs of the smartphone screen]

A photo of the app as it appeared on the phones of those in the social feedback group is shown in Fig. 1, and an example of the on-screen feedback, in Fig. 2. (The screens seen by those in the individual feedback group were very similar.) Participants could view feedback on their phones at any time of day and the data was refreshed every fifteen minutes. This feedback was provided in a number of formats. When the app was first opened, participants in the intervention groups were shown a running total of the number of steps they had taken that day, along with an estimate of the calories they had burnt by taking those steps and of the number of miles that they had walked. They then had the option of viewing equivalent data for the previous day or viewing ‘past week’ or ‘history’ screens. These last two screens used line graphs that allowed easy comparison of steps taken on different days, with the latter permitting users to use a swipe action to switch between data displays of different weeks. Those in the social feedback group were also able to compare their data to the average for other participants in that group.

The study ran for eight weeks between October and December, with data from the mobile phones automatically downloaded to a secure central server. The intention had been for the first two weeks to provide baseline data, but due to a technical malfunction, data from this ‘run in’ phase was unusable.

Standardised text messages were sent to participants throughout the trial (see Table 1). In weeks 1–2, four messages reminded all participants to carry their phones in their pockets. In week 3, those in the treatment groups received messages on the Monday the app was activated and all three groups were sent a message on the Thursday. Subsequently, those in the two experimental groups were sent weekly messages to encourage them to walk more and those in the control group were messaged once a fortnight to remind them to carry their phones. Participants were sent the following message if their phones had not sent data for one or two days: “[First name], we’ve not had any data from your app for [1, 2] days. After 3 days, we might have to withdraw you from the research and ask for the phone back. Text, call or email if you need any help.” No participants were excluded from the study for this reason.

**Table 1:** Examples of the SMS messages sent to participants during the trial

	Total number	Example 1: 24^th^ Oct; day 1, week 1	Example 2: day 1, week 3	Example 3: day 4, week 3	Example 4: day 4, week 5
Control group	9	[First name], thank you for taking part in this important research! Except when doing sport, please keep the phone in your trouser pocket from now on.	no message	In the bag? That’s a snag! Remember to keep your phone in your pocket!	We’re half-way through the study. Many thanks!
Individual group	16		Your bActive app is now fully activated. Please open it and take some time to explore and use it.	Walking is one of the best activities for your health. How much are you doing? Check the app!	To improve your fitness, ‘brisk is best’. But it is a good idea to use the ‘talk test’: can you talk while you walk?
Social group	16			Walking is one of the best activities for your health. Are you doing more than others, or less? Check the app!	

Participants were provided with a participant information sheet and written consent was obtained from all participants. The study was not registered with a research register, although we recognise that doing so is increasingly emerging as best practice. Ethical approval was provided by Swansea University’s research ethics committee.

The data collected in this study had a two-level structure: step-counts were collected over a series of days (first level) but were also clustered within participants (second level). For this reason, analysis was conducted using multilevel regression models (MLM), an established methodology in public health research [34]. MLM has a range of advantages over other methods [35]. Any attempt to understand behaviour without taking account of data hierarchies can severely handicap explication of the underlying processes [36] because inter-observation dependency in the data can lead to the underestimation of standard errors of regression coefficients and an overstatement of statistical significance. MLM avoids this problem by partitioning the within- and between-subjects variance of the dependent variable. In addition, unlike in a repeated measures ANOVA, only the missing observations themselves are deleted if data are missing for any time point. The final advantage of MLM is that it facilitates the easy fitting of within-subjects auto-correlation, and thereby acknowledges the sequential repeated collection of observations from the same subject.

In this study, MLM was implemented using a four-step analytical process. First, an unconditional model was run – i.e., a model with no predictors, just partitioning of within-subject and between-subject variance. The Model Deviance statistic of this model provided a baseline fit against which subsequent models could be tested. It also enabled the assessment of the variance to be explained at the within-subject and between-subject levels. Finally, it allowed calculation of the percentage of variance in the outcome that was attributable to differences between subjects (the ICC-1 statistic).

The second step was to fit a fixed-effects growth model in order to examine and control the shape and direction of any change over time in the number of steps taken per day. Linear and quadratic effects of time were therefore added as predictors. To control for any association of step-counts with weekly behaviour patterns, a dummy variable was created for day of the week.

The third step was a test for variability, between the participants, in changes in the step-count. This was achieved by allowing the coefficients of the growth parameters (the linear and squared effects of time) to vary between subjects. The final step was the addition of the effects of the *Experimental Group* (defined as both interventions groups together) and its interaction with time-point. This tested whether the *Experimental Group* accounted for any variation in the intercept level or any change in the outcome variable. The following variables were controlled for at this stage: marital status, number of children in the household age sixteen or under, employment status, ownership of a motorised vehicle or bicycle, and previous ownership of a smartphone.

At each of the stages, model improvement was evaluated by testing the reduction in the model deviance and assessing the extra variance explained. Within-subjects auto-correlation was modelled using an AR1-type correlation matrix. To negate the distorting effects of the handful of participants that had very large numbers of steps in any one day, the outcome variable was log-transformed.

## Results

**Fig. 3:**
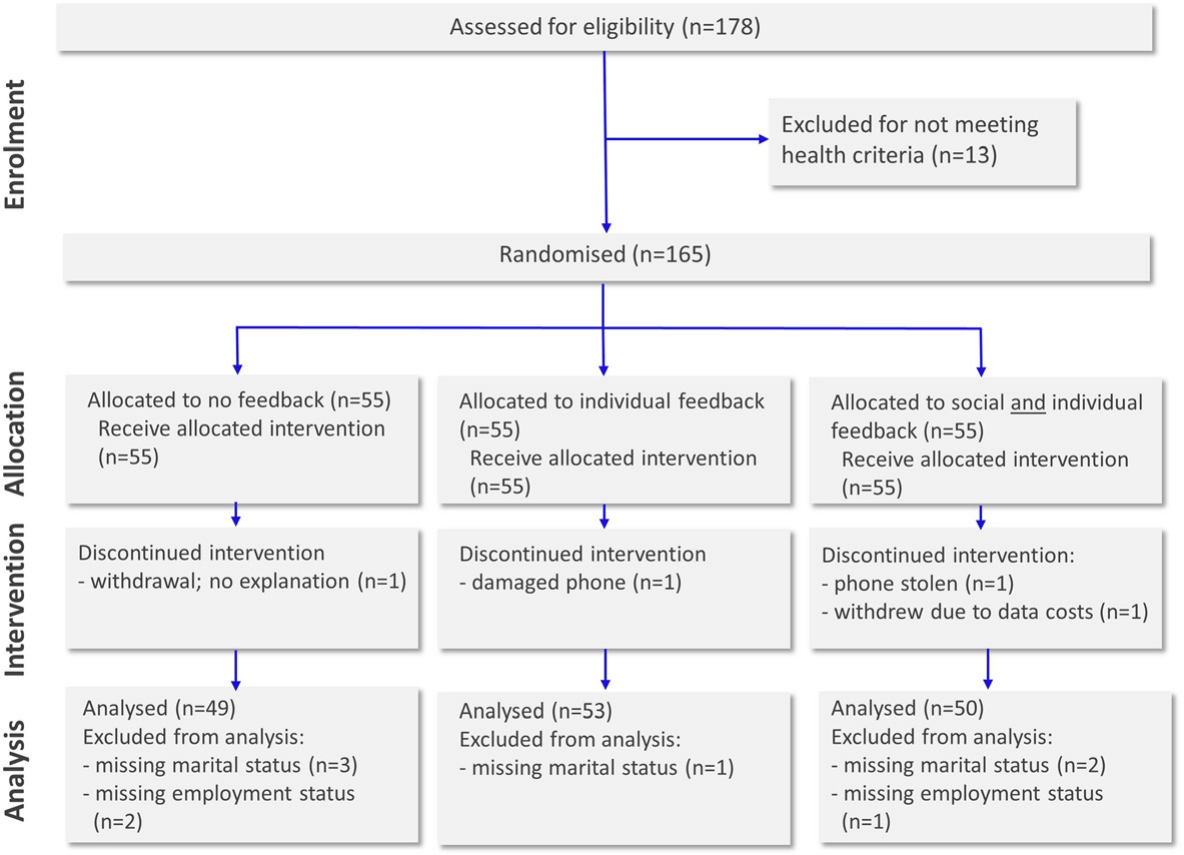
The flow diagram of bActive study. [This image provides a flow diagram of the study]

Of the 165 original recruits, 161 participants completed the study in its entirety. No participants were excluded from the study for non-compliance. Nine participants were excluded from the statistical analysis because of missing demographic data. Two were unable to complete because their phone was damaged or stolen, one withdrew without giving a reason and one gave data costs as the reason for withdrawal. There was no statistically significant relationships between those who dropped out, or were excluded from the analysis, and step-counts or the experimental group. The characteristics of the 152 participants included in the analysis are summarised in Table 2. A flow diagram is provided in Fig. 3.

**Table 2:** Demographic information of participants

	Total (*n* =152)		Control group (*n* = 49)	Individual feedback group (*n* = 53)	Social feedback group (*n* = 50)
Marital/family status					
- single no children	70	46 %	25	24	21
- single with children	6	4 %	0	3	3
- with partner, no children	40	26 %	11	14	15
- with partner and children	29	19 %	10	10	9
- other	7	4 %	3	2	2
Employment status					
- full-time employed/self-employed	104	69 %	32	35	37
- part-time employed	13	9 %	2	7	4
- carer/unemployed	14	8 %	6	3	3
- student	21	14 %	8	7	6
Type of employment					
- sedentary (e.g., office worker)	58	50 %	25	20	13
- moderately active (e.g., teacher)	51	44 %	8	19	24
- very active (e.g., postal worker)	7	6 %	1	3	3
Regularly participate in sport	89	59 %	31	29	29
Own motorised transport	95	63 %	30	31	34
Previously owned a smartphone	108	72 %	36	38	34

**Fig. 4:**
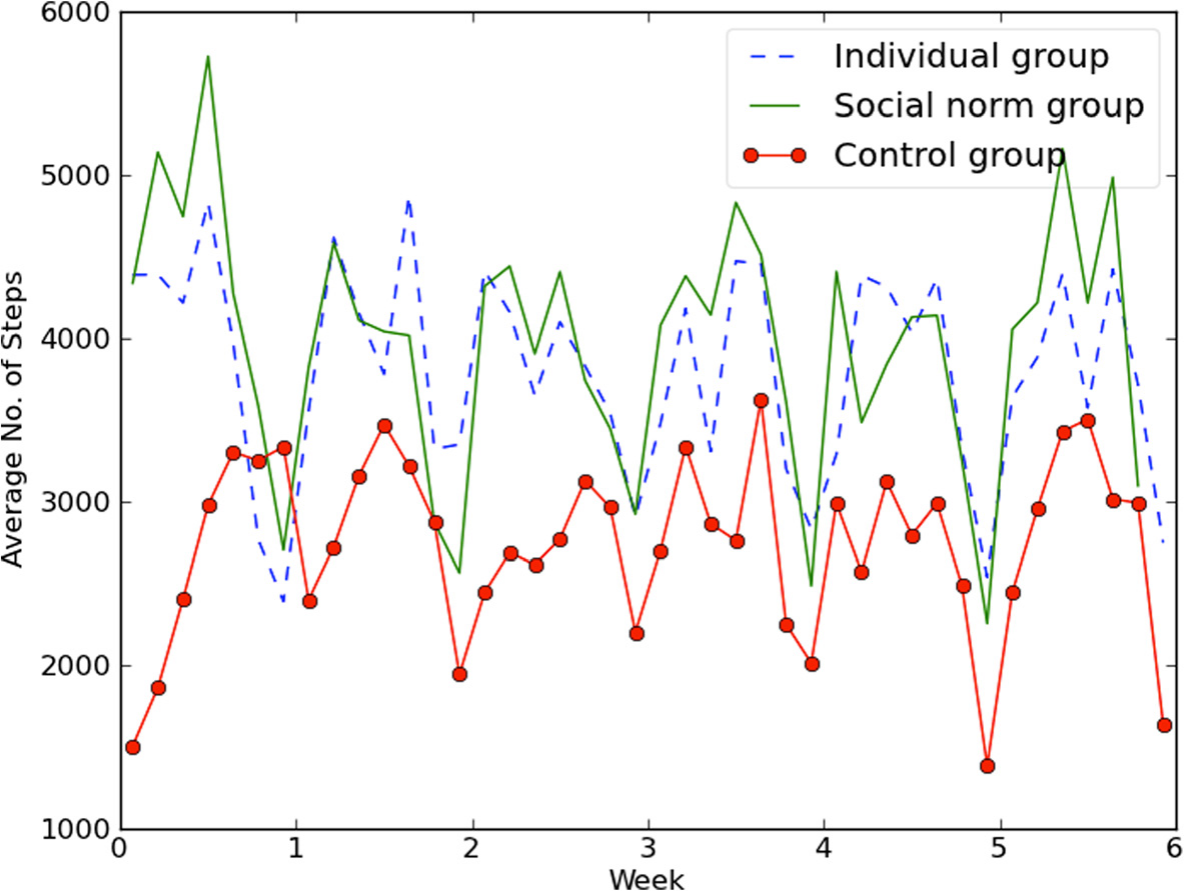
Results plotted over the six-weeks of the trail for the control group and two treatment groups. [This image provides a graphical display of the daily number of steps taken by the three groups in the study]

A total of 6214 observations were recorded over 42 days across the 152 subjects, with 92 % of the subjects (i.e., all but 13) providing observations on at least 40 days. Average daily levels of recorded activity were higher in the individual and social feedback groups (3842 and 3984 steps, respectively) than in the control group (2822 steps). Information on the average number of steps taken by participants in each treatment group is provided in Table 3; Fig. 4 visualises this data, showing the week-by-week variation in average step-counts for weeks 1 to 6 of the intervention.

**Table 3:** Average numbers of steps recorded over the 6-week trial

Day of the week	Control (*n* = 49)			Individual feedback group (*n* = 53)			Social feedback group (*n* = 50)			Complete sample (*n* = 152)		
	Mean	Median	SD	Mean	Median	SD	Mean	Median	SD	Mean	Median	SD
Monday	2399	1373	2837	3848	2772	3989	4181	2714	4252	3495	2348	3829
Tuesday	2651	1548	2937	4344	3196	4168	4320	3023	4498	3796	2653	4011
Wednesday	2955	1940	2986	4066	3178	3640	4285	2955	4296	3781	2884	3722
Thursday	3182	2030	3097	4197	3477	3916	4539	3150	4501	3980	3005	3919
Friday	3293	2142	3502	4371	3058	4654	4318	3170	4068	4010	2808	4149
Saturday	2756	1727	3373	3322	2153	3519	3289	2144	3566	3132	1978	3494
Sunday	2080	973	2842	2811	1749	3474	2452	1676	2697	2462	1411	3052

As one would expect, the variation in steps showed a high level of clustering within participants. However, an ICC (1) statistic of 0.33 indicated that a third of the total variation was due to between-participant differences. The introduction of linear and quadratic effects of time (i.e., modelling change over time in individuals’ walking habits) alongside dummy codes for day of the week explained a statistically significant, but small (4 %), within-participant variance. Tests of fixed effects coefficients indicated that of the three predictors (the linear effect of time, the quadratic (curvilinear) effect of time and day of the week), the third was the primary explanatory variable (see Fig. 4); this suggests that within-individual variation in walking was also due to variation in daily routine. Since modelling of curvilinear change offered no improvement over a simple linear effect, the quadratic effect of time was dropped from the model.

There was evidence that the linear effect of time varied between individuals. When this random effect was added, along with the covariance between starting level and extent of linear change, the model deviance reduced significantly (SD = 111 on 2df, *p* < 0.0005) and the unexplained within-participant variance was reduced by a further 4 %. Of the demographic and control variables, only employment status and car ownership had a significant effect on step counts, with full-time and part-time employees likely to have a higher step count than other groups and car owners likely to have lower step counts than non-car owners.

The tests for hypotheses H1 and H3 show that *Experimental Group* (a dummy variable with control group as the reference category) had a statistically significant effect on step-count (F = 6.626, *p* < 0.0005). Furthermore, adding Experimental Group to the model significantly reduced its deviance (change in deviance = 13 on 2df, *p* < 0.0005) and explained a further 7.7 % of the between-participants variance in step-count. The coefficients for differences between the individual and social feedback groups vs. the control group were both statistically significant (individual vs. control B = 0.474; 95 % CI = 0.166–0.782; *p* < 0.05, and social vs. control B = 0.526; 95 % CI = 0.212–0.840; *p* < 0.05). When the log-transformed outcomes were transformed back, these coefficients gave an expected step-count 60 % higher for those in the individual group (exponential(0.474) = 1.60) and 69 % higher for the social group (exponential(0.526) = 1.69). The null hypothesis was therefore rejected for both H1 and H3 in favour of the alternative: that those receiving either form of feedback had higher step-counts than those in the control group. However, there was no statistically significant difference in step-count between the two experimental groups, so the null hypothesis for H2 could not be rejected.

The interaction of *Experimental Group* and time-point was not statistically significant, so the effect of individual and social feedback on step-count was immediate and did not increase or decrease across the study period. The rate and direction of change in step-count over the study period did not vary significantly according to *Experimental Group*: adding this interaction effect reduced model deviance by just 1 on 1df (*p* > 0.05) and explained only 0.6 % of the variation in slopes.

## Discussion

This study indicates that always-on, accelerometer-based smartphone apps can generate a substantial increase in walking amongst relatively healthy, young to early-middle-aged men. These behaviour changes were independent of marital or employment status, whether there are children in the home and ownership of motorised transport. This suggests that this approach may successfully be applied to population segments that currently fail to meet physical activity targets [37, 38]. As technology makes working and domestic life increasingly sedentary [39], such interventions can alert people to their levels of inactivity and prompt them to counter this change with subtle changes to their daily practices. In addition, the bActive approach minimises conscious cognitive effort rather than eliminating it, and influences both the behaviour and the understanding of participants [40]. As a result, unlike the nudges delivered by Thaler and Sunstein’s [41] libertarian paternalism, it cannot be criticised for being manipulative and non-reflective.

Furthermore, the intervention reported here relied solely on automated feedback displays and easily-automated motivational text messages. Unlike interventions used in many previous population-level studies [42–44], it did not rely on a programme of expensive face-to-face support, motivation or instruction such. Hence, it can be delivered easily and affordably to large populations.

Some comment is needed on the larger than expected differences in step-counts between the feedback groups and the control. It is possible that Bristol’s reputation as a ‘green’ city may have made users more amenable to extra walking than men in other UK areas and that the scale of the change is not therefore generalizable. However, as reported elsewhere [38], use of the app had a transformative effect on some users, leading them to attribute greater benefit to the walking that was part of their normal activities and to recognise that they could become more physically active simply by changing the way they organised existing everyday practices. As strategies for increasing their step-counts, participants in the feedback groups reported, for example making additional trips to the shops, taking dogs for longer walks and going to see colleagues when they would previously have sent them emails. These behaviour changes resulted, it appears, from an awareness, facilitated by the app, of long sedentary periods within their daily lives and the number of steps inherent in simple, day-to-day practices (or variations of practices) that they had not formerly associated with physical activity. There are many potential sources of variation in physical activity including seasonal factors, emotional factors, support from others, weather, and competing demands on time.

Although qualitative interviews conducted ten months after trial-end suggest that some participants still had a raised level of physical activity [14], it is likely that some of the more outlandish behaviours (e.g., walking round the house simply in order to reach step targets) will have ceased with time. No data was available to test long-term outcomes for the sample as a whole because the length of this trial was constrained by the burden placed on participants by the need to forgo the use of their usual phones and carry the study phone with them at all times. Future studies will need to find a way around this problem.

There is also the question of whether, when they were not obliged to do so, users’ intrinsic motivation to carry the phone with them would be sufficient to provide sufficient data to prompt behaviour change. Previously published evidence from this trial suggests that it would be [14]. It reported that feedback participants opened the app an average 3.9 times per day, each time keeping it open for an average of 32.0 s, and that in the final week they were still opening it 2.3 times a day. Furthermore, 91 % described the app as ‘interesting’, 67 % as ‘fun’ and 73 % said they would continue to use the app after the trial.

Ultimately, the ideal target population for this app would be people who are at risk of adverse health outcomes related to inactivity. Some of those in this category might have no more than a passing interest in becoming more active. This is why the intervention was designed to require minimal commitment (the app runs automatically), minimal financial investment (no additional devices are needed) and to promote engagement through simple curiosity about the feedback rather than through any particular desire to exercise. The app used in this study has two main strengths in this regard: being on continuously, it measures the walking inherent in practices not usually considered as ‘exercise’, and it required neither the purchase of specialised equipment, commitment to an exercise regime or self-identification as fitness-conscious and exercise-oriented. As a result, it is likely to have greater appeal to this population than many of the pedometer-style systems that are currently available and, therefore, to be more successful at changing their walking behaviours.

The use of the approach for both men and women remains problematic however. New technology does allow step-data to be collected by devices that do not need to be located in any particular part of the body and that could more easily be carried by those not wearing trousers with pockets. However, the costs of purchasing additional electronic devices would undermine the scalability of an intervention, and the need to carry an extra piece of equipment might deter some of the population being targeted. Furthermore, the focus on men does have some justification: although a range of apps have previously been developed specifically for female users [24, 27, 45], few studies have explored the effects on males of using this type of approach.

A key finding of this research was the lack of significant incremental effect related to social norm feedback. This unexpected outcome appears to contradict the literature on social norms, which argues that normative comparisons significantly enhance the impact of behavioural feedback – including in health-related behaviours such as substance abuse [19, 41]. An alternative interpretation, however, is that this finding indicates issues with the design of the social feedback used. Practitioners of the social norms approach argue that the most effective reference group consists of those that participants consider most like themselves [23, 46, 47]. Although all participants were of the same gender and of approximately the same age, the social feedback might have been more effective if the age-band had been narrower or if a distinction had been made between, for example, those in physically active occupations and those doing more sedentary work. Alternatively, the social feedback may have distracted users from the individual feedback, thereby masking the incremental impact of the former.

Work is needed to explore the effectiveness of this type of intervention for other parts of the population, including older men and people with health conditions that were excluded by the screening used in this study. Although the sample included people who were not inclined to exercise, research is also needed that focuses exclusively on this population. Future studies should assess the extent to which changes in walking behaviour are sustained over time.

## Conclusion

This study provides evidence that the techniques used in this app significantly increase physical activity levels in male adults. For those in this category not actively seeking to become more active, the minimal requirements for commitment (the app runs automatically) and financial investment (no additional devices are needed) should be an advantage, as too should the lack of any need to commit to a physical activity regime or self-identify as fitness-conscious and exercise-oriented. Another advantage for this group is the promotion of engagement through simple curiosity and the absence of any reliance on a desire to do more physical activity. Being on continuously, the app measures the walking inherent in practices not usually considered as ‘exercise’. This, it has been found [38], piques the curiosity of users and provides encouragement to those who were not previously aware that day-to-day walking could be seen in this way [48, 49]. The app is therefore likely to have greater appeal to this population than many of the pedometer-style systems that are currently available and to be more successful at changing their walking behaviours.
